# What, where, and when: the importance of post-transcriptional regulation in the brain

**DOI:** 10.3389/fnins.2013.00192

**Published:** 2013-10-29

**Authors:** Michael A. Kiebler, Peter Scheiffele, Jernej Ule

**Affiliations:** ^1^Department for Anatomy and Cell Biology, Ludwig Maximilian UniversityMunich, Germany; ^2^Biozentrum, University of BaselBasel, Switzerland; ^3^Department of Molecular Neuroscience, University College London Institute of NeurologyLondon, UK

**Keywords:** alternative splicing, RNA editing, RNA methylation, translation, mRNA trafficking, axon guidance, synaptic plasticity, neurological disease

## Introduction

Until recently, RNA metabolism has been considered a purely academic topic that kept a small group of molecular biologists busy. Thanks to many emerging techniques—mainly, but not exclusively—systems approaches that employ high-throughput sequencing, such as UV cross-linking and immunoprecipitation (CLIP), ribosome profiling and RNAseq, we gained major new insights into the importance of RNA metabolism for brain function, as well as malfunction (Darnell, 2013). In that context, the brain presents a particularly fascinating diversity of post-transcriptional gene regulation through many new and recently discovered mechanisms. Neurons exhibit remarkably rich molecular repertoires that match their complex morphologies and functions. RNA is often localized to various subcompartments in order to exert specific local functions. Moreover, dynamic changes in RNA processing and turnover provide powerful mechanisms for neuronal plasticity. The central importance of these mechanisms is highlighted by the severe neurological disorders associated with defects in post-transcriptional processing functions in the brain.

## Emerging techniques

The last decade marked an expansion of genome-wide experimental and computational techniques that provide unprecedented insights into the mechanisms and physiological relevance of post-transcriptional regulation in the brain, and how it can go awry in disease (Darnell, 2013; Modic et al., 2013). The new methods enable to study protein-RNA and miRNA-RNA interactions with high specificity and positional resolution (Gascon and Gao, 2012; Konig et al., 2012). Moreover, the development of RNAseq, ribosome profiling and related emerging functional genomic and computational methods, has enabled global studies of alternative splicing, RNA editing, methylation, stability and translation (Ingolia et al., 2009; Norris and Calarco, 2012; Tariq and Jantsch, 2012; Trivedi and Deth, 2012).

## Molecular diversity for diverse cellular functions

A remarkable feature of neuronal and glial cells is their morphological and functional diversity. Such specialized functions are achieved through highly complex gene expression programs. The brain exhibits the highest levels of alternative splicing and RNA editing (Norris and Calarco, 2012; Tariq and Jantsch, 2012). Even though functions of individual alternative protein isoforms are understood for only a few cases, it is clear that intact regulation of alternatively splicing is required for the development of neurons or glia, and for the formation of functional synapses (Norris and Calarco, 2012). Moreover, A to I editing often alters the critical properties of neuronal receptors and channels, and is thereby required for synaptic transmission (Tariq and Jantsch, 2012). Thus, pre-mRNA processing and editing greatly enhances proteome diversity, and thus the functional complexity of the nervous system.

## mRNA transport and local translation

In addition to molecular diversity, post-transcriptional mechanisms also are key contributors to spatial-temporal control of neuronal mRNA functions. In neuronal precursor cells, RNA localization is required for asymmetric divisions of neuronal progenitor cells. Knockdown of certain key RBP regulators of mRNA localization causes premature differentiation of radial glial cells into neurons (Kusek et al., 2012; Vessey et al., 2012). Later on, control of mRNA translation or degradation can take place within neuronal axons and dendrites, due to the unique ability of neurons to transport mRNAs far from the cell body. Local translation of mRNAs within axonal growth cones or within dendritic spines enables neurons to remodel these critical structures. Thus, the local proteome and function of neuronal sub-compartments can be acutely and selectively modified in response to specific signals. This enables rapid and selective control of processes such as axon guidance and synaptic plasticity at sites that are remote from the cell body.

A recent study identified as many as 2550 transcripts that are transported to either axons or dendrites (Cajigas et al., 2012). mRNA transport and local translation depend on cis-acting regulatory elements that are recognized by RBPs, forming a ribonucleoprotein complex (RNP) that directs mRNA transport and translation (Doyle and Kiebler, 2011). In navigating axons, RNPs control the choice of mRNAs that are translated in response to extrinsic cues, which in turn determines the direction of axon growth (Hornberg and Holt, 2013). Similarly, mRNA transport to neuronal dendrites is controlled by specific cis-acting elements. Here, Tongiorgi and colleagues (Baj et al., 2013) present a hypothesis suggesting how a common single nucleotide polymorphism in the human brain-derived neurotrophic factor gene (*BDNF*) gene may affect the dendritic transport of BDNF mRNA, and thereby cause deficits in memory. This remains an area of intense research, with a recent study suggesting an anterograde, rather than retrograde mode of BDNF action (Dieni et al., 2012).

## Synaptic plasticity

mRNAs localized to dendrites have a key function in synaptic plasticity. In response to synaptic stimuli, local control of mRNA translation near synapses is required to facilitate long-lasting forms of synaptic plasticity, the cellular basis for learning, and memory formation (Kapeli and Yeo, 2012; Fernandez et al., 2013). This does not only involve local control of mRNA polyadenylation and translation, but also protein degradation via the proteasome (Cajigas et al., 2010; Udagawa et al., 2012). Moreover, all aspects of mRNA regulation, from nuclear RNA editing to local control of mRNA translation, play crucial roles in the alteration of the synaptic proteome that is required to maintain synaptic homeostasis and prevent pathological recurrent network excitation (Turrigiano, 2011; Penn et al., 2013). In this context, a new hypothesis is being proposed for the methyltransferase PRMT, which is regulated by redox status and can methylate the RGG domain of RBPs such as FUS, which could modulate regulatory functions of RNPs and thereby affect synaptic function (Trivedi and Deth, 2012).

## Altered RNA metabolism in disease

Mutations in RBPs, toxic RNA repeats, or other defects in post-transcriptional regulation contribute to a variety of neurologic diseases, especially motor neuron diseases (Ramaswami et al., 2013). This is corroborated by changes in pre-mRNA processing or RNA editing of important neuronal receptors or channels, which were observed neurodegenerative and psychiatric disorders, as well as epilepsy (Tariq and Jantsch, 2012). Moreover, it was recently proposed that specific RBPs or miRNAs might be secreted from stressed motoneurons to stimulate defence mechanisms in astrocytes or endothelial cells (Aparicio-Erriu and Prehn, 2012; Gascon and Gao, 2012). This indicates that perturbed homeostasis of RBPs or miRNAs, and the consequent changes in RNA metabolism may play a central role in neurodegenerative processes (Aparicio-Erriu and Prehn, 2012; Gascon and Gao, 2012; Kapeli and Yeo, 2012).

Taken together, we feel that the present collection of reviews on the mRNA life cycle in normal brain function and malfunction provides a timely update by leading researchers to reflect recent developments in key technologies, and summarizes the current understanding and future directions for the studies of mRNA metabolism in the brain.
